# 1-Cyclo­hexyl­sulfinyl-2-methyl­naphtho­[2,1-*b*]furan

**DOI:** 10.1107/S1600536811010981

**Published:** 2011-03-31

**Authors:** Hong Dae Choi, Pil Ja Seo, Byeng Wha Son, Uk Lee

**Affiliations:** a Department of Chemistry, Dongeui University, San 24 Kaya-dong Busanjin-gu, Busan 614-714, Republic of Korea; b Department of Chemistry, Pukyong National University, 599-1 Daeyeon 3-dong, Nam-gu, Busan 608-737, Republic of Korea

## Abstract

In the title compound, C_19_H_20_O_2_S, the cyclo­hexyl ring adopts a chair conformation and the aryl­sulfinyl unit is positioned equatorial relative to the cyclo­hexyl group. In the crystal, mol­ecules are linked through weak inter­molecular C—H⋯O hydrogen bonds. The O atom of the sulfinyl group is disordered over two orientations with site-occupancy factors of 0.923 (3) and 0.077 (3).

## Related literature

For the pharmacological activity of naphtho­furan compounds, see: Einhorn *et al.* (1984 ▶); Hranjec *et al.* (2003 ▶); Mahadevan & Vaidya (2003 ▶). For structural studies of related 2-methyl­naphtho­[2,1-*b*]furan derivatives, see: Choi *et al.* (2006 ▶, 2007 ▶).
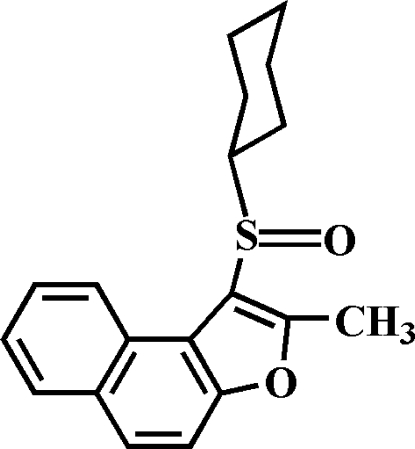


## Experimental

### 

#### Crystal data

C_19_H_20_O_2_S
*M* *_r_* = 312.41
Monoclinic, 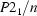

*a* = 5.8424 (1) Å
*b* = 19.5900 (3) Å
*c* = 13.4314 (2) Å
β = 92.649 (1)°
*V* = 1535.62 (4) Å^3^
*Z* = 4
Mo *K*α radiation
μ = 0.22 mm^−1^
*T* = 173 K
0.42 × 0.34 × 0.26 mm

#### Data collection

Bruker SMART APEXII CCD diffractometer
Absorption correction: multi-scan (*SADABS*; Bruker, 2009 ▶) *T*_min_ = 0.916, *T*_max_ = 0.946
14260 measured reflections
3527 independent reflections
3026 reflections with *I* > 2σ(*I*)
*R*_int_ = 0.037

#### Refinement

*R*[*F* ^2^ > 2σ(*F* ^2^)] = 0.041
*wR*(*F* ^2^) = 0.104
*S* = 1.06
3527 reflections
210 parameters
4 restraints
H-atom parameters constrained
Δρ_max_ = 0.56 e Å^−3^
Δρ_min_ = −0.49 e Å^−3^

### 

Data collection: *APEX2* (Bruker, 2009 ▶); cell refinement: *SAINT* (Bruker, 2009 ▶); data reduction: *SAINT*; program(s) used to solve structure: *SHELXS97* (Sheldrick, 2008 ▶); program(s) used to refine structure: *SHELXL97* (Sheldrick, 2008 ▶); molecular graphics: *ORTEP-3* (Farrugia, 1997 ▶) and *DIAMOND* (Brandenburg, 1998 ▶); software used to prepare material for publication: *SHELXL97*.

## Supplementary Material

Crystal structure: contains datablocks global, I. DOI: 10.1107/S1600536811010981/fl2340sup1.cif

Structure factors: contains datablocks I. DOI: 10.1107/S1600536811010981/fl2340Isup2.hkl

Additional supplementary materials:  crystallographic information; 3D view; checkCIF report

## Figures and Tables

**Table 1:** Hydrogen-bond geometry (Å, °)

*D*—H⋯*A*	*D*—H	H⋯*A*	*D*⋯*A*	*D*—H⋯*A*
C13—H13*B*⋯O2*B*^i^	0.98	2.12	2.848 (6)	130
C14—H14⋯O2*A*^ii^	1.00	2.38	3.2947 (19)	152

Symmetry codes: (i) 
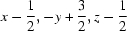
; (ii) 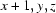.

**Fig. 1.**
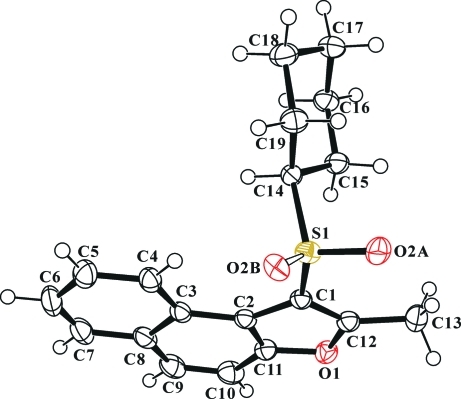
The molecular structure of the title compound with the atom numbering scheme. Displacement ellipsoids are drawn at the 50% probability level. H atoms are presented as a small spheres of arbitrary radius. The O atom of sulfinyl group is disordered over two positions with site-occupancy factors, from refinement of 0.923 (3) (part A) and 0.077 (3) (part B).

**Fig. 2.**
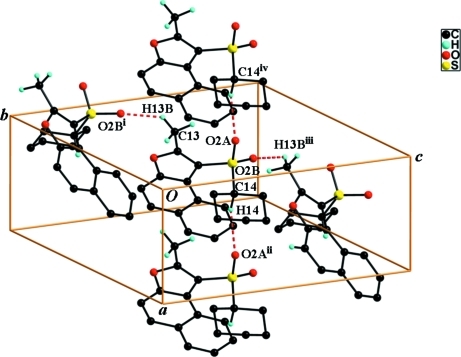
A view of the C—H···O hydrogen bonds (dotted lines) in the crystal structure of the title compound. [Symmetry codes: (i) x - 1/2, -y + 3/2,z - 1/2; (ii) x + 1, y, z; (iii) x + 1/2, - y + 3/2, z + 1/2 ; (iv) x - 1, y, z.]

## supplementary crystallographic information

### Comment

Many compounds containing a naphthofuran ring have attracted much attention owing to their potent pharmacological properties such as antibacterial, antitumor and anthelmintic activities (Einhorn *et al.* , 1984, Hranjec *et al.*, 2003, Mahadevan & Vaidya, 2003). As a part of our ongoing studies of the substituent effect on the solid state structures of 2-methylnaphtho[2,1-b]furan analogues (Choi *et al.*, 2006, 2007), we report herein the crystal structure of the title compound.

In the title molecule (Fig. 1), the naphthofuran unit is essentially planar, with a mean deviation of 0.014 (1) Å from the least-squares plane defined by the thirteen constituent atoms. The cyclohexyl ring is in the chair form and arylsulfinyl moiety is positioned equatorial relative to the cyclohexyl group. The O atom of the sulfinyl group is disordered over two positions with site-occupancy factors, from refinement, of 0.923 (3) (part A) and 0.077 (3) (part B). The crystal packing (Fig. 2) is stabilized by weak intermolecular C—H···O hydrogen bonds; the first one between a methyl H atom and and one of the disordered sulfinyl oxygen atoms(Table 1; C13—H13B···O2B^i^), and the second one between a cyclohexyl H atom and the other disordered sulfinyl oxygen (Table 1; C14—H14···O2A^ii^).

### Experimental

77% 3-chloroperoxybenzoic acid (291 mg, 1.3 mmol) was added in small portions to a stirred solution of 1-cyclohexylsulfanyl-2-methylnaphtho[2,1-b] furan (355 mg, 1.2 mmol) in dichloromethane (40 mL) at 273 K. After being stirred at room temperature for 8h, the mixture was washed with a saturated sodium bicarbonate solution and the organic layer was separated, dried over magnesium sulfate, filtered and concentrated at reduced pressure. The residue was purified by column chromatography (hexane-ethyl acetate, 4:1 v/v) to afford the title compound as a colorless solid [yield 74%, m.p. 429–430 K; *R*_f_ = 0.61 (hexane–ethyl acetate, 4:1 v/v)]. Single crystals suitable for X-ray diffraction were prepared by slow evaporation frroomm acetone at room temperature

### Refinement

All H atoms were positioned geometrically and refined using a riding model, with C—H = 0.95 Å for aryl, 1.00 Å for methine, 0.99 Å for methylene and 0.98 Å for methyl H atoms, respectively. *U*_iso_(H) =1.2*U*_eq_(C) for aryl, methine and methylene, and 1.5*U*_eq_(C) for methyl H atoms. The O atom of sulfinyl group is disordered over two positions with site-ccupancy factors, from refinement of 0.923 (3) (part A) and 0.077 (3) (part B). The S—O distances were restrained to be within 0.001 Å of one another using the SADI and DELU commands.

### Crystal data

**Table:**

C_19_H_20_O_2_S	*F*(000) = 664
*M**_r_* = 312.41	*D*_x_ = 1.351 Mg m^−^^3^
Monoclinic, *P*2_1_/*n*	Mo *K*α radiation, λ = 0.71073 Å
Hall symbol: -P 2yn	Cell parameters from 6431 reflections
*a* = 5.8424 (1) Å	θ = 2.6–27.5°
*b* = 19.5900 (3) Å	µ = 0.22 mm^−^^1^
*c* = 13.4314 (2) Å	*T* = 173 K
β = 92.649 (1)°	Block, colourless
*V* = 1535.62 (4) Å^3^	0.42 × 0.34 × 0.26 mm
*Z* = 4	

### Data collection

**Table:**

Bruker SMART APEXII CCD diffractometer	3527 independent reflections
Radiation source: rotating anode	3026 reflections with *I* > 2σ(*I*)
graphite multilayer	*R*_int_ = 0.037
Detector resolution: 10.0 pixels mm^-1^	θ_max_ = 27.5°, θ_min_ = 1.8°
φ and ω scans	*h* = −7→7
Absorption correction: multi-scan (*SADABS*; Bruker, 2009)	*k* = −21→25
*T*_min_ = 0.916, *T*_max_ = 0.946	*l* = −17→13
14260 measured reflections	

### Refinement

**Table:**

Refinement on *F*^2^	Primary atom site location: structure-invariant direct methods
Least-squares matrix: full	Secondary atom site location: difference Fourier map
*R*[*F*^2^ > 2σ(*F*^2^)] = 0.041	Hydrogen site location: difference Fourier map
*wR*(*F*^2^) = 0.104	H-atom parameters constrained
*S* = 1.06	*w* = 1/[σ^2^(*F*_o_^2^) + (0.0439*P*)^2^ + 0.6943*P*] where *P* = (*F*_o_^2^ + 2*F*_c_^2^)/3
3527 reflections	(Δ/σ)_max_ = 0.001
210 parameters	Δρ_max_ = 0.56 e Å^−^^3^
4 restraints	Δρ_min_ = −0.49 e Å^−^^3^

### Special details

**Table:**

Geometry. All esds (except the esd in the dihedral angle between two l.s. planes) are estimated using the full covariance matrix. The cell esds are taken into account individually in the estimation of esds in distances, angles and torsion angles; correlations between esds in cell parameters are only used when they are defined by crystal symmetry. An approximate (isotropic) treatment of cell esds is used for estimating esds involving l.s. planes.
Refinement. Refinement of F^2^ against ALL reflections. The weighted R-factor wR and goodness of fit S are based on F^2^, conventional R-factors R are based on F, with F set to zero for negative F^2^. The threshold expression of F^2^ > 2sigma(F^2^) is used only for calculating R-factors(gt) etc. and is not relevant to the choice of reflections for refinement. R-factors based on F^2^ are statistically about twice as large as those based on F, and R- factors based on ALL data will be even larger.

### Fractional atomic coordinates and isotropic or equivalent isotropic displacement parameters (Å^2^)

**Table:**

	*x*	*y*	*z*	*U*_iso_*/*U*_eq_	Occ. (<1)
S1	0.41951 (7)	0.69277 (2)	0.70702 (3)	0.02494 (12)	
O1	0.37126 (19)	0.80010 (6)	0.45636 (8)	0.0279 (3)	
O2A	0.1985 (2)	0.65645 (7)	0.69667 (10)	0.0324 (4)	0.923 (3)
O2B	0.428 (3)	0.7216 (7)	0.8085 (4)	0.032 (4)	0.077 (3)
C1	0.4458 (3)	0.74856 (8)	0.60361 (11)	0.0217 (3)	
C2	0.6184 (3)	0.80058 (8)	0.59149 (11)	0.0223 (3)	
C3	0.8144 (3)	0.82508 (8)	0.64850 (12)	0.0240 (3)	
C4	0.8863 (3)	0.80032 (9)	0.74330 (12)	0.0275 (4)	
H4	0.8002	0.7656	0.7738	0.033*	
C5	1.0795 (3)	0.82587 (10)	0.79219 (13)	0.0349 (4)	
H5	1.1264	0.8085	0.8559	0.042*	
C6	1.2078 (3)	0.87735 (10)	0.74869 (15)	0.0394 (5)	
H6	1.3410	0.8947	0.7831	0.047*	
C7	1.1428 (3)	0.90261 (9)	0.65739 (15)	0.0363 (4)	
H7	1.2316	0.9375	0.6288	0.044*	
C8	0.9452 (3)	0.87778 (8)	0.60411 (13)	0.0287 (4)	
C9	0.8795 (3)	0.90357 (9)	0.50783 (14)	0.0346 (4)	
H9	0.9706	0.9381	0.4796	0.042*	
C10	0.6905 (3)	0.88046 (9)	0.45488 (14)	0.0338 (4)	
H10	0.6470	0.8981	0.3909	0.041*	
C11	0.5645 (3)	0.82935 (8)	0.49987 (12)	0.0261 (3)	
C12	0.3020 (3)	0.75066 (8)	0.52147 (12)	0.0245 (3)	
C13	0.0955 (3)	0.71211 (9)	0.48597 (13)	0.0312 (4)	
H13A	0.1376	0.6791	0.4351	0.047*	
H13B	−0.0190	0.7439	0.4572	0.047*	
H13C	0.0315	0.6878	0.5421	0.047*	
C14	0.6408 (3)	0.63113 (8)	0.67666 (11)	0.0218 (3)	
H14	0.7919	0.6552	0.6773	0.026*	
C15	0.5947 (3)	0.59934 (8)	0.57445 (12)	0.0266 (3)	
H15A	0.6037	0.6350	0.5226	0.032*	
H15B	0.4381	0.5799	0.5702	0.032*	
C16	0.7685 (3)	0.54320 (9)	0.55551 (13)	0.0298 (4)	
H16A	0.7305	0.5217	0.4901	0.036*	
H16B	0.9232	0.5635	0.5530	0.036*	
C17	0.7697 (3)	0.48904 (9)	0.63642 (14)	0.0337 (4)	
H17A	0.8868	0.4541	0.6232	0.040*	
H17B	0.6184	0.4663	0.6359	0.040*	
C18	0.8223 (3)	0.52122 (9)	0.73821 (13)	0.0331 (4)	
H18A	0.9786	0.5408	0.7403	0.040*	
H18B	0.8175	0.4856	0.7904	0.040*	
C19	0.6489 (3)	0.57741 (9)	0.75944 (12)	0.0294 (4)	
H19A	0.4950	0.5569	0.7647	0.035*	
H19B	0.6920	0.5995	0.8239	0.035*	

### Atomic displacement parameters (Å^2^)

**Table:**

	*U*^11^	*U*^22^	*U*^33^	*U*^12^	*U*^13^	*U*^23^
S1	0.0245 (2)	0.0235 (2)	0.0274 (2)	0.00162 (15)	0.00739 (15)	0.00115 (15)
O1	0.0266 (6)	0.0291 (6)	0.0277 (6)	0.0003 (5)	−0.0021 (5)	0.0036 (5)
O2A	0.0207 (7)	0.0343 (8)	0.0425 (8)	−0.0019 (5)	0.0062 (5)	0.0037 (6)
O2B	0.030 (8)	0.022 (8)	0.045 (7)	0.003 (6)	0.003 (6)	0.014 (6)
C1	0.0208 (7)	0.0193 (7)	0.0251 (7)	0.0015 (6)	0.0029 (6)	−0.0013 (6)
C2	0.0225 (7)	0.0188 (7)	0.0259 (7)	0.0019 (6)	0.0042 (6)	−0.0017 (6)
C3	0.0224 (8)	0.0200 (7)	0.0297 (8)	0.0003 (6)	0.0039 (6)	−0.0065 (6)
C4	0.0264 (8)	0.0275 (8)	0.0287 (8)	−0.0014 (7)	0.0018 (6)	−0.0066 (6)
C5	0.0320 (9)	0.0392 (10)	0.0331 (9)	0.0013 (8)	−0.0027 (7)	−0.0119 (8)
C6	0.0266 (9)	0.0409 (11)	0.0504 (11)	−0.0073 (8)	−0.0003 (8)	−0.0200 (9)
C7	0.0291 (9)	0.0293 (9)	0.0510 (11)	−0.0079 (7)	0.0080 (8)	−0.0116 (8)
C8	0.0272 (8)	0.0211 (8)	0.0384 (9)	−0.0012 (7)	0.0078 (7)	−0.0060 (7)
C9	0.0369 (10)	0.0245 (9)	0.0434 (10)	−0.0058 (7)	0.0112 (8)	0.0024 (7)
C10	0.0381 (10)	0.0280 (9)	0.0356 (9)	0.0004 (7)	0.0058 (8)	0.0076 (7)
C11	0.0245 (8)	0.0242 (8)	0.0297 (8)	0.0014 (6)	0.0018 (6)	−0.0004 (6)
C12	0.0235 (8)	0.0229 (8)	0.0274 (8)	0.0024 (6)	0.0033 (6)	−0.0010 (6)
C13	0.0258 (8)	0.0359 (10)	0.0315 (9)	−0.0028 (7)	−0.0015 (7)	−0.0034 (7)
C14	0.0192 (7)	0.0203 (7)	0.0260 (7)	0.0001 (6)	0.0020 (6)	−0.0012 (6)
C15	0.0295 (8)	0.0243 (8)	0.0260 (8)	0.0041 (7)	−0.0007 (6)	−0.0015 (6)
C16	0.0317 (9)	0.0251 (8)	0.0327 (9)	0.0044 (7)	0.0023 (7)	−0.0043 (7)
C17	0.0328 (9)	0.0227 (8)	0.0454 (10)	0.0048 (7)	0.0009 (8)	0.0005 (7)
C18	0.0338 (9)	0.0292 (9)	0.0362 (9)	0.0051 (7)	−0.0013 (7)	0.0074 (7)
C19	0.0314 (9)	0.0289 (9)	0.0279 (8)	0.0019 (7)	0.0023 (7)	0.0034 (7)

### Geometric parameters (Å, °)

**Table:**

S1—O2B	1.4741 (16)	C10—C11	1.397 (2)
S1—O2A	1.4752 (13)	C10—H10	0.9500
S1—C1	1.7796 (16)	C12—C13	1.483 (2)
S1—C14	1.8287 (15)	C13—H13A	0.9800
O1—C11	1.372 (2)	C13—H13B	0.9800
O1—C12	1.3782 (19)	C13—H13C	0.9800
C1—C12	1.356 (2)	C14—C15	1.520 (2)
C1—C2	1.448 (2)	C14—C19	1.530 (2)
C2—C11	1.377 (2)	C14—H14	1.0000
C2—C3	1.431 (2)	C15—C16	1.526 (2)
C3—C4	1.408 (2)	C15—H15A	0.9900
C3—C8	1.431 (2)	C15—H15B	0.9900
C4—C5	1.374 (2)	C16—C17	1.519 (2)
C4—H4	0.9500	C16—H16A	0.9900
C5—C6	1.400 (3)	C16—H16B	0.9900
C5—H5	0.9500	C17—C18	1.524 (3)
C6—C7	1.360 (3)	C17—H17A	0.9900
C6—H6	0.9500	C17—H17B	0.9900
C7—C8	1.416 (2)	C18—C19	1.532 (2)
C7—H7	0.9500	C18—H18A	0.9900
C8—C9	1.424 (3)	C18—H18B	0.9900
C9—C10	1.363 (3)	C19—H19A	0.9900
C9—H9	0.9500	C19—H19B	0.9900
			
O2B—S1—O2A	105.2 (6)	C12—C13—H13A	109.5
O2B—S1—C1	119.1 (6)	C12—C13—H13B	109.5
O2A—S1—C1	109.24 (8)	H13A—C13—H13B	109.5
O2B—S1—C14	117.8 (6)	C12—C13—H13C	109.5
O2A—S1—C14	106.57 (7)	H13A—C13—H13C	109.5
C1—S1—C14	98.29 (7)	H13B—C13—H13C	109.5
C11—O1—C12	106.44 (12)	C15—C14—C19	111.88 (13)
C12—C1—C2	107.20 (14)	C15—C14—S1	111.98 (11)
C12—C1—S1	125.51 (12)	C19—C14—S1	106.89 (10)
C2—C1—S1	127.28 (12)	C15—C14—H14	108.7
C11—C2—C3	119.04 (14)	C19—C14—H14	108.7
C11—C2—C1	104.89 (14)	S1—C14—H14	108.7
C3—C2—C1	136.07 (15)	C14—C15—C16	110.75 (13)
C4—C3—C2	124.48 (15)	C14—C15—H15A	109.5
C4—C3—C8	118.85 (15)	C16—C15—H15A	109.5
C2—C3—C8	116.67 (15)	C14—C15—H15B	109.5
C5—C4—C3	120.71 (16)	C16—C15—H15B	109.5
C5—C4—H4	119.6	H15A—C15—H15B	108.1
C3—C4—H4	119.6	C17—C16—C15	111.43 (14)
C4—C5—C6	120.46 (18)	C17—C16—H16A	109.3
C4—C5—H5	119.8	C15—C16—H16A	109.3
C6—C5—H5	119.8	C17—C16—H16B	109.3
C7—C6—C5	120.37 (17)	C15—C16—H16B	109.3
C7—C6—H6	119.8	H16A—C16—H16B	108.0
C5—C6—H6	119.8	C16—C17—C18	110.31 (14)
C6—C7—C8	121.19 (17)	C16—C17—H17A	109.6
C6—C7—H7	119.4	C18—C17—H17A	109.6
C8—C7—H7	119.4	C16—C17—H17B	109.6
C7—C8—C9	121.08 (16)	C18—C17—H17B	109.6
C7—C8—C3	118.42 (16)	H17A—C17—H17B	108.1
C9—C8—C3	120.49 (16)	C17—C18—C19	110.89 (14)
C10—C9—C8	122.26 (16)	C17—C18—H18A	109.5
C10—C9—H9	118.9	C19—C18—H18A	109.5
C8—C9—H9	118.9	C17—C18—H18B	109.5
C9—C10—C11	116.30 (16)	C19—C18—H18B	109.5
C9—C10—H10	121.9	H18A—C18—H18B	108.1
C11—C10—H10	121.9	C14—C19—C18	110.95 (13)
O1—C11—C2	111.06 (14)	C14—C19—H19A	109.5
O1—C11—C10	123.70 (15)	C18—C19—H19A	109.5
C2—C11—C10	125.22 (16)	C14—C19—H19B	109.5
C1—C12—O1	110.39 (14)	C18—C19—H19B	109.5
C1—C12—C13	135.26 (15)	H19A—C19—H19B	108.0
O1—C12—C13	114.34 (14)		
			
O2B—S1—C1—C12	−129.3 (7)	C12—O1—C11—C2	−0.83 (17)
O2A—S1—C1—C12	−8.48 (16)	C12—O1—C11—C10	177.79 (15)
C14—S1—C1—C12	102.38 (15)	C3—C2—C11—O1	−179.51 (13)
O2B—S1—C1—C2	49.3 (7)	C1—C2—C11—O1	1.20 (17)
O2A—S1—C1—C2	170.18 (13)	C3—C2—C11—C10	1.9 (2)
C14—S1—C1—C2	−78.96 (14)	C1—C2—C11—C10	−177.40 (16)
C12—C1—C2—C11	−1.11 (17)	C9—C10—C11—O1	−179.38 (15)
S1—C1—C2—C11	−179.96 (12)	C9—C10—C11—C2	−1.0 (3)
C12—C1—C2—C3	179.78 (17)	C2—C1—C12—O1	0.64 (17)
S1—C1—C2—C3	0.9 (3)	S1—C1—C12—O1	179.53 (10)
C11—C2—C3—C4	179.47 (15)	C2—C1—C12—C13	179.33 (17)
C1—C2—C3—C4	−1.5 (3)	S1—C1—C12—C13	−1.8 (3)
C11—C2—C3—C8	−1.3 (2)	C11—O1—C12—C1	0.08 (17)
C1—C2—C3—C8	177.71 (16)	C11—O1—C12—C13	−178.90 (13)
C2—C3—C4—C5	178.74 (15)	O2B—S1—C14—C15	173.0 (7)
C8—C3—C4—C5	−0.5 (2)	O2A—S1—C14—C15	55.20 (13)
C3—C4—C5—C6	0.3 (3)	C1—S1—C14—C15	−57.80 (12)
C4—C5—C6—C7	−0.1 (3)	O2B—S1—C14—C19	50.1 (7)
C5—C6—C7—C8	0.0 (3)	O2A—S1—C14—C19	−67.66 (12)
C6—C7—C8—C9	−179.00 (17)	C1—S1—C14—C19	179.35 (11)
C6—C7—C8—C3	−0.1 (3)	C19—C14—C15—C16	−54.42 (18)
C4—C3—C8—C7	0.3 (2)	S1—C14—C15—C16	−174.40 (11)
C2—C3—C8—C7	−178.92 (14)	C14—C15—C16—C17	56.20 (18)
C4—C3—C8—C9	179.24 (15)	C15—C16—C17—C18	−57.71 (19)
C2—C3—C8—C9	0.0 (2)	C16—C17—C18—C19	57.25 (19)
C7—C8—C9—C10	179.84 (17)	C15—C14—C19—C18	54.40 (18)
C3—C8—C9—C10	1.0 (3)	S1—C14—C19—C18	177.31 (12)
C8—C9—C10—C11	−0.5 (3)	C17—C18—C19—C14	−55.59 (19)

### Hydrogen-bond geometry (Å, °)

**Table:**

*D*—H···*A*	*D*—H	H···*A*	*D*···*A*	*D*—H···*A*
C13—H13B···O2B^i^	0.98	2.12	2.848 (6)	130
C14—H14···O2A^ii^	1.00	2.38	3.2947 (19)	152

Symmetry codes: (i) *x*−1/2, −*y*+3/2, *z*−1/2; (ii) *x*+1, *y*, *z*.
